# Unified Space–Time-Message Interference Alignment: An End-to-End Learning Approach

**DOI:** 10.3390/e28020249

**Published:** 2026-02-21

**Authors:** Elaheh Sadeghabadi, Steven Blostein

**Affiliations:** Department of Electrical and Computer Engineering, Queen’s University, Kingston, ON K7L 3N6, Canada; steven.blostein@queensu.ca

**Keywords:** broadcast channel, end-to-end design, interference alignment, imperfect channel state information, common message decoding

## Abstract

This paper investigates the performance of a multi-user multiple-input single-output (MU-MISO) broadcast channel under the practical constraints of imperfect, delayed, and quantized channel state information at the transmitter (CSIT). Conventional interference alignment (IA) strategies—classified into spatial (SIA), temporal (TIA), and message-domain (MIA) techniques— typically designed for specific, idealized CSI regimes and often rely on successive interference cancellation (SIC) at the receiver. However, the iterative structure of SIC is highly susceptible to error propagation, particularly under CSI uncertainty and high-order modulation. We propose Deep-STMIA, a novel end-to-end deep learning framework that jointly optimizes interference management across the space, time, and message domains. Using a neural network-based autoencoder architecture with structural message-domain regularization, Deep-STMIA learns to mitigate the catastrophic effects of error propagation and adapts to a continuum of CSIT conditions. Simulation results demonstrate that Deep-STMIA matches the performance of degrees-of-freedom (DoF) optimal benchmarks in extreme CSI regimes and significantly outperforms state-of-the-art baselines, such as rate-splitting multiple access (RSMA), in practical imperfect CSIT scenarios.

## 1. Introduction

The performance of multi-user broadcast channel (BC) systems is fundamentally limited by multi-user interference and the quality of channel state information at the transmitter (CSIT). In recently emerging standards for wireless communications, such as the new 5G radio (NR) and the emerging 6G framework, effective interference management is crucial to support massive connectivity and high-reliability requirements, yet the signaling overhead required for perfect CSIT remains a major barrier to maximizing spectral efficiency [1].

In a frequency-division duplexing (FDD) system, the channel state information (CSI) is typically estimated by the receivers and then fed back to the base station (BS). This feedback process introduces overhead and imperfections in the CSIT. To reduce feedback, each receiver typically quantizes its channel direction vector using a distinct codebook of size $2^{b_{f}}$, where $b_{f}$ is the number of feedback bits. This process is analogous to quantization in lossy source coding, where the codebook represents the set of quantization levels. Consequently, the transmitter only has an estimated and quantized version of the true CSI [2,3,4].

In addition to quantization, propagation and processing delays exist in the feedback loop. That is, the CSIT is often delayed and outdated relative to the current channel state. For channels that vary rapidly over time, i.e., those with low temporal correlation, this delay severely degrades received CSIT quality. In summary, a multi-user multiple-input single-output broadcast channel (MU-MISO BC) system operates with imperfect, delayed, and quantized CSIT. This three-fold system imperfection results in partial CSIT operation. Performance degradation for these systems is typically characterized in terms of achievable rate and degrees of freedom (DoF) [5].

The current approach to mitigating multi-user interference, especially that created by imperfect CSIT, lies in the design of advanced interference alignment (IA) techniques [6,7]. A core IA design principle is to pre-process transmitted signals so that the interference caused at each receiver is confined to a subspace of smaller dimension than the total signal space. This strategy creates interference-free dimensions for the desired signal reception. Current IA techniques can be broadly classified into three domains:

- Space-Domain Interference Alignment (SIA) manages interference by designing precoding vectors based on the available CSIT to direct beams from a multi-antenna transmitter. In a MISO BC with perfect CSIT, zero forcing (ZF) [8] or dirty paper coding (DPC) [9] techniques aim to completely eliminate interference, achieving the maximum sum DoF of $\min\left(M,K\right)$ for *M* transmit antennas and *K* single-antenna users.
- Time-Domain Interference Alignment (TIA) leverages the channel’s temporal dimension by *retrospective* IA design over multiple time intervals. A notable example is the Maddah-Ali and Tse (MAT) scheme [10], which uses perfect delayed CSIT to send an interference-resolving signal in a subsequent interval to resolve interference from the past. TIA has been shown to be highly effective in time-correlated channels [11,12].
- Message-Domain Interference Alignment (MIA) manages interference by encoding a common message stream intended for all users in addition to private user messages. This approach, typically implemented through rate-splitting multiple access (RSMA) [1,5,11,13], is particularly robust to imperfect CSIT [14], which has been identified as a key enabler for various 6G scenarios, ranging from satellite communications to massive MIMO [15]. RSMA allocates the interference components most vulnerable to CSIT inaccuracy to the common stream. Since the common message is designed for universal decodability, it remains robust against CSI uncertainty, protecting private streams from the catastrophic interference typical of conventional multi-user precoding [5,13,16].

When both delayed and current channel information are available, a combined space–time interference alignment (STIA) scheme can be designed to strategically utilize both spatial and temporal dimensions [11,12]. The effective integration of SIA, TIA, and MIA is essential to maximize performance in environments characterized by imperfect CSIT.

Most existing schemes proposed to handle imperfect CSIT rely on theoretical, system-level metrics such as DoF. While DoF analysis provides a metric for the asymptotic high signal-to-noise ratio (SNR) regime, it often fails to capture the practical complexities and non-linear interactions present in real-world systems. Link-level simulations, on the other hand, better capture finite SNR regimes, specific modulation and coding schemes, and realistic channel models. An objective of this paper is to bridge the gap between theoretical system-level analysis and practical link-level performance by considering an end-to-end (E2E) communication system model.

To approach global optimality, E2E systems are currently implemented using deep learning (DL)-based autoencoders, which offer a powerful alternative to model-based designs [17,18,19]. This data-driven approach allows for the joint optimization of the entire communication chain, enabling the learning of efficient, non-linear strategies tailored specifically to complex and imperfect channel conditions.

In the following, we propose an E2E DL-based framework for MU-MISO BC, called *deep space–time-message interference alignment (Deep-STMIA)*. The main contributions are as follows:

- A novel deep-STMIA framework based on E2E DL is proposed to jointly optimize interference alignment across the spatial, temporal, and message domains.
- Unlike conventional schemes tailored to specific CSIT regimes, the proposed approach exhibits generalized CSI robustness and learns a unified communication strategy that adapts to a continuum of CSIT conditions—ranging from no CSIT and delayed CSIT to imperfect and perfect current CSIT.
- In contrast to traditional modular designs, recent advances in semantic communications [20] suggest that joint optimization of the message representation and the physical layer transmission can significantly enhance reliability. The proposed Deep-STMIA framework adopts this philosophy by regularizing the message domain to ensure that the transmitted streams are inherently robust to the interference patterns of the space-time channel. In particular, a structural message-domain regularization mechanism is proposed using auxiliary common bits, enabling the network to autonomously perform common message decoding for interference management and mitigate CSIT uncertainty. This allows the framework to improve upon RSMA for uncertainty-prone channels.
- By replacing rigid, iterative processing with successive interference cancellation (SIC), the Deep-STMIA neural network approach implicitly mitigates decoding bottlenecks from catastrophic error propagation, particularly for high-order modulation and imperfect CSIT scenarios.
- Validation and performance gains are demonstrated by extensive simulations. The reliability of Deep-STMIA, measured via block error rate (BLER), is observed to match the performance slopes of DoF-optimal benchmarks in extreme CSIT regimes and significantly outperform existing RSMA [1] and time-correlated [11] schemes in imperfect CSIT conditions.

The remainder of this paper is organized as follows: Section 2 details the system model for the MU-MISO BC with imperfect CSIT, which incorporates common message decoding. Section 3 provides the background for three IA schemes and highlights examples in the literature. Section 4 presents the proposed Deep-STMIA architecture and its implementation with the integrated IA schemes. Section 5 discusses the simulation setup and results. Finally, Section 6 concludes the paper.

**Notation:** Bold upper- and lower-case letters denote matrices and vectors, respectively. $\left|M\right|$ is the cardinality of set M. The conjugate, transpose, and conjugate-transpose are ${\left(\cdot \right)}^{*}$, ${\left(\cdot \right)}^{T}$, and ${\left(\cdot \right)}^{H}$, respectively. R and C represent real and complex fields. $x\overset{d}{∼}\mathrm{CN}\left(\mu ,Q\right)$ denotes that x is a circularly symmetric complex Gaussian distribution. The notations $\forall n=n_{1},\ldots ,n_{2}$ and ${\left\{\cdot \right\}}_{n=n_{1}}^{n_{2}}$ represent integers from $n_{1}$ to $n_{2}$. The *n*-th element of a vector x is represented by ${\left[x\right]}_{n}$. E{·} denotes expectation. The projection matrix on the direction x is $\Psi \left(x\right)=\frac{xx^{H}}{{∥x∥}^{2}}$, and $x^{⊥}$ is any non-zero vector such that $x^{H}x^{⊥}=0$. *a*∼*b* denotes that *a* scales with *b* in the asymptotic regime.

## 2. System Model

Consider an MU-MISO BC system comprised of a single BS equipped with *M* transmit antennas and *K* single-antenna users. Communication occurs over *T* time intervals, where the time interval t∈{1,…,T} consists of $n_{t}$ channel uses.

**Figure 1:**
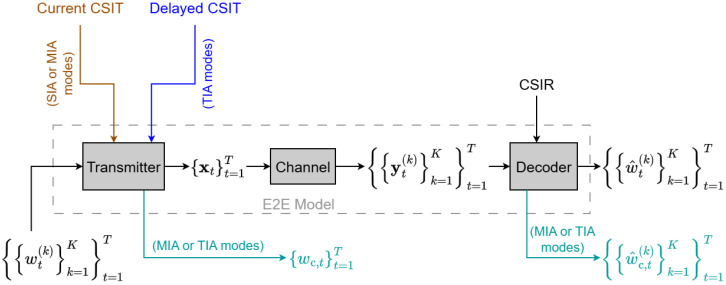
The proposed Deep-STMIA E2E architecture for MU-MISO BC is represented, where the inputs and outputs of the transmitter and decoder blocks depend on the IA techniques applied (SIA, MIA, and TIA modes). The Decoder block includes *K* receivers.

At the beginning of each time interval *t*, the system transmits a set of private messages and a common message. The private message intended for user *k* at time *t* is denoted by $w_{t}^{\left(k\right)}$, carrying $b_{t}^{\left(k\right)}$ information bits, for all k=1,…,K. To facilitate TIA and/or MIA, the transmitter generates a common message, $w_{c,t}$, in each time interval *t*, containing $b_{c,t}$ bits, which must be decoded by all users, as shown in Figure 1.

The common message $w_{c,t}$ and the private messages ${\left\{w_{t}^{\left(k\right)}\right\}}_{k=1}^{K}$ are jointly encoded in the transmitted signal $x_{t}\in C^{Mn_{t}}$ for the $n_{t}$ channel uses. The *n*-th symbol transmitted at time *t* is $x_{t,n}\in C^{M}$, where $x_{t}={\left[x_{t,1}^{T},\ldots ,x_{t,n_{t}}^{T}\right]}^{T}$. The average transmit power constraint per-symbol is:

(1) $$\begin{array}{c} E\left\{∥x_{t,n}{∥}^{2}\right\}\leq 1,\;\forall n=1,\ldots ,n_{t},\forall t=1,\ldots ,T. \end{array}$$

The channel vector for user *k* in the *n*-th channel use of the time interval *t* is $h_{t,n}^{\left(k\right)}\in C^{M}$. The channel vectors for all users are concatenated into matrix $H_{t,n}\in C^{K\times M}$. The signal received by user *k* during the time interval *t* is $y_{t}^{\left(k\right)}={\left[y_{t,1}^{\left(k\right)},\ldots ,y_{t,n_{t}}^{\left(k\right)}\right]}^{T}\in C^{n_{t}}$, where the signal received by user *k* in the *n*-th channel use of the *t*-th time interval $y_{t,n}^{\left(k\right)}$ is given by

(2) $$\begin{array}{cc} y_{t,n}^{\left(k\right)} & ={h_{t,n}^{\left(k\right)}}^{H}x_{t,n}+\epsilon_{t,n}^{\left(k\right)},\;\forall n=1,\ldots ,n_{t},\forall t=1,\ldots ,T,\forall k=1,\ldots ,K, \end{array}$$

where $\epsilon_{t,n}^{\left(k\right)}\overset{d}{∼}\mathrm{CN}\left(0,\frac{1}{P}\right)$ is additive white Gaussian noise (AWGN) with independent and identical distribution (i.i.d.) and *P* is the SNR. Upon receiving $y_{t}^{\left(k\right)}$, the user *k* attempts to estimate the common decoded message, denoted by ${\hat{w}}_{c,t}^{\left(k\right)}$, and estimate its private message, denoted by ${\hat{w}}_{t}^{\left(k\right)}$.

The full instantaneous channel vector $h_{t,n}^{\left(k\right)}$ is modeled as the sum of an estimated component, ${\hat{h}}_{t,n}^{\left(k\right)}$, and unknown error component ${\overset{ˇ}{h}}_{t,n}^{\left(k\right)}$:

(3) $$\begin{array}{c} h_{t,n}^{\left(k\right)}={\hat{h}}_{t,n}^{\left(k\right)}+{\overset{ˇ}{h}}_{t,n}^{\left(k\right)}. \end{array}$$

Both components are modeled as complex Gaussian random vectors with respective covariance matrices

(4) $$\begin{array}{cc} E\left\{{\hat{h}}_{t,n}^{\left(k\right)}{\hat{h}}_{t,n}^{{\left(k\right)}^{H}}\right\} & =\left(1-\sigma^{2}\right)I_{M},\;\mathrm{and} \end{array}$$

(5) $$\begin{array}{cc} E\left\{{\overset{ˇ}{h}}_{t,n}^{\left(k\right)}{\overset{ˇ}{h}}_{t,n}^{{\left(k\right)}^{H}}\right\} & =\sigma^{2}I_{M}. \end{array}$$

Parameter $\sigma^{2}$ quantifies the quality of the CSI estimate and is modeled by the function $\sigma^{2}=P^{-\alpha_{P}}$, controlled by parameter $\alpha_{P}$. Of interest is the asymptotic regime (P→∞), where the exponent $\alpha =\lim_{P\to \infty }\alpha_{P}$ dictates the CSI quality: α=1 for near-perfect CSI and α=0 for no CSI knowledge. Here, $\alpha_{P}=\frac{\alpha P}{P+1-\alpha }$, for SNR *P*. This function satisfies the asymptotic behavior of $\alpha_{P}$ mentioned above and ensures that the effective quality is bounded within [0,1] and monotonically increases with *P*, as encountered in practical situations where CSI estimation error is noise-limited.

To model varying levels of channel knowledge, three specific CSI scenarios for the *n*-th channel use of time interval *t* are defined. Each scenario follows the Gaussian error model in (3)–(5), with the error variance determined by a specific quality exponent:

- Local CSIR (${\left\{{\left\{{\bar{H}}_{t,n}\right\}}_{n=1}^{n_{t}}\right\}}_{t=1}^{T}$): Each user possesses local channel knowledge with quality parameter γ. The error variance for CSIR is $\sigma_{R}^{2}=P^{-\gamma_{P}}$, where $\gamma_{P}=\frac{\gamma P}{P+1-\gamma }$.
- Delayed CSIT (${\left\{{\left\{{\tilde{H}}_{t,n}\right\}}_{n=2}^{n_{t}}\right\}}_{t=1}^{T}$): The transmitter receives past channel states with quality parameter β. The corresponding error variance is $\sigma_{D}^{2}=P^{-\beta_{P}}$, where $\beta_{P}=\frac{\beta P}{P+1-\beta }$. This delayed feedback enables TIA.
- Current CSIT (${\left\{{\left\{{\hat{H}}_{t,n}\right\}}_{n=1}^{n_{t}}\right\}}_{t=1}^{T}$): The transmitter possesses instantaneous channel knowledge with quality parameter α. The error variance is $\sigma_{C}^{2}=P^{-\alpha_{P}}$, where $\alpha_{P}$ is defined above. Current CSIT is essential for SIA and MIA.

Parameters {α,β,γ}∈[0,1] represent asymptotic CSI quality as P→∞. Based on inherent physical layer considerations, including limited feedback and delay, the CSI quality parameters are assumed to satisfy the following hierarchy:

(6) $$\begin{array}{c} \gamma \geq \beta \geq \alpha . \end{array}$$

The above relationship states that CSI estimated locally at the receiver (with quality parameter γ) is the most accurate, and that the quality of the delayed CSIT (β) is greater than the quality of the current CSIT (α). It is also assumed that the current CSIT is estimated from the delayed CSIT by taking into account the temporal correlation in the channel.

## 3. DoF Optimal Baseline Schemes

This section provides a brief review of the optimal DoF-achieving schemes for the two-user MISO BC under different CSIT assumptions. These schemes serve as baselines for performance evaluation of the proposed E2E DL approach and illustrate the utility of the SIA, TIA, and MIA techniques as a function of CSIT quality.

The literature on MIMO BC under the imperfect CSIT described above can be characterized by the exponent α, where the CSIT error variance $\sigma^{2}$∼$P^{-\alpha }$:

1. Perfect Current CSIT (α=1): Enables full space-domain IA (SIA), such as ZF precoding.
2. No CSIT (α=0): Requires orthogonal schemes, such as time division multiple access (TDMA).
3. Perfect Delayed CSIT and No Current CSIT (β=1 and α=0) [10]: Enables time-domain IA (TIA), such as the MAT scheme.
4. Imperfect Current CSIT (0<α<1) [2,4,11,16]: Enables a combination of SIA and message-domain IA (MIA) using rate-splitting (RS).
5. Perfect Delayed CSIT and Imperfect Current CSIT (β=1 and 0<α<1) [11]: Enables a joint space–time IA (STIA) scheme using both current and delayed CSIT.

Another category exists for imperfect delayed CSIT and imperfect current CSIT [11,21]. This case is ignored because, as shown in [11], when delayed CSIT is imperfect, applying retrospective IA can potentially reduce the overall DoF.

The fundamental performance limit for two-user MISO BC with M=2 antennas is defined by its achievable DoF region. According to [11] (Theorem 1), the optimal DoF region for a two-user BC with perfect delayed CSIT and imperfect current CSIT characterized by $P^{-\alpha }$, is bounded by the following:

(7) $$\begin{array}{c} \left\{\begin{array}{cc} d_{1} & \leq 1,\\ d_{2} & \leq 1,\\ d_{1}+2d_{2} & \leq 2+\alpha ,\\ 2d_{1}+d_{2} & \leq 2+\alpha . \end{array}\right. \end{array}$$

The non-trivial vertices of this region are $\left(\alpha ,1\right)$, $\left(1,\alpha \right)$, and $\left(\frac{2+\alpha }{3},\frac{2+\alpha }{3}\right)$. The achievement of these vertices demonstrates the optimality of the schemes.

**Figure 2:**
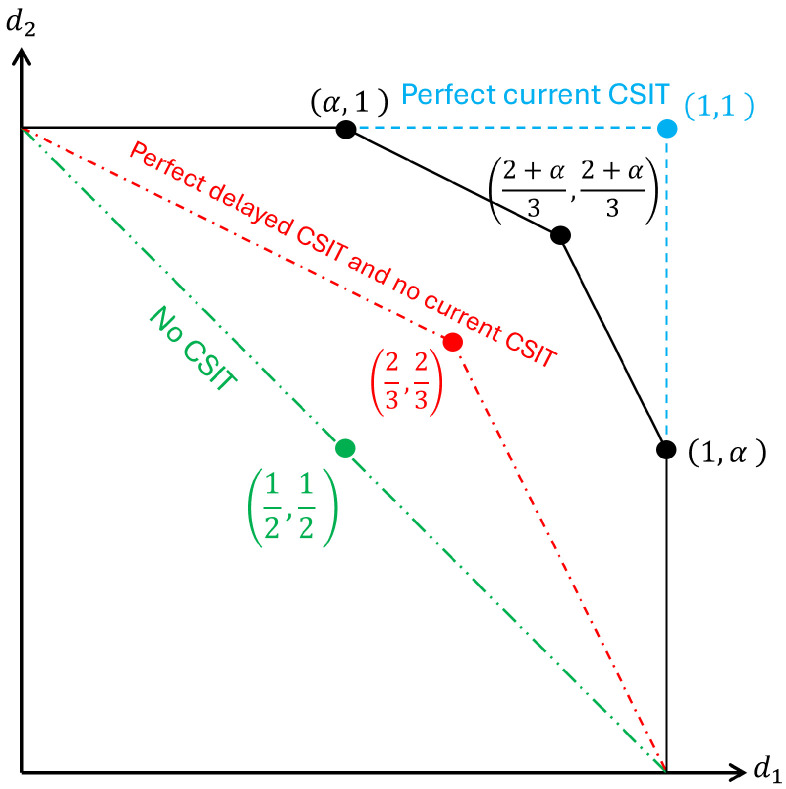
The DoF region of the two-user MISO system under various CSIT assumptions. The regions for delayed and imperfect CSIT are adapted from [11] (Figure 1), while the No-CSIT baseline is added to illustrate the relationship in Equation (7).

Figure 2 illustrates the DoF region, where the following hold:

- The green region corresponds to the minimum case of no CSIT (α=0).
- The blue region corresponds to the maximum DoF case of perfect CSIT (α=1).
- The red region corresponds to the specific case of perfect delayed CSIT and no current CSIT (α=0).
- The black region represents the general case of 0≤α≤1.

Table 1 summarizes the sum-DoF achieved by the schemes indicated above at specific DoF points, which serve as the optimal baselines under their respective CSIT conditions. In Section 3.1, Section 3.2 and Section 3.3, which follow next, assume that the messages for users 1 and 2, $w_{1}^{\left(1\right)}$ and $w_{1}^{\left(2\right)}$, are encoded into codewords $u={\left[u_{1}^{T},\ldots ,u_{\bar{n}}^{T}\right]}^{T}$ and $v={\left[v_{1}^{T},\ldots ,v_{\bar{n}}^{T}\right]}^{T}$, respectively, where $u_{n},v_{n}\in C^{M},\forall n=1,\ldots ,\bar{n},$ are part of the user codewords sent in the *n*-th channel use.

### 3.1. Zero Forcing

With perfect current CSIT (α=1), the ZF precoding technique is used to completely mitigate multi-user interference. This relies purely on SIA.

The scheme spans one time interval (T=1). The transmitted signal is $x_{1}=u+v$, where the precoding is such that $Q_{u_{n}}=E\left\{u_{n}u_{n}^{H}\right\}=\frac{P}{2}\Psi \left({h_{1,n}^{\left(2\right)}}^{⊥}\right)$ and $Q_{v_{n}}=E\left\{v_{n}v_{n}^{H}\right\}=\frac{P}{2}\Psi \left({h_{1,n}^{\left(1\right)}}^{⊥}\right),\forall n=1,\ldots ,n_{1}$ for $n_{1}=\bar{n}$, where Ψ(x) is the projection matrix in the direction x, i.e., $\Psi \left(x\right)=\frac{xx^{H}}{{∥x∥}^{2}}$. The precoding ensures that the unintended user’s channel vector is orthogonal to the intended user’s precoded signal. The received signals for $n_{1}=n$ are as follows:

(8) $$\begin{array}{cc} y_{1,n}^{\left(1\right)} & ={h_{1,n}^{\left(1\right)}}^{H}u_{n}+\epsilon_{1,n}^{\left(1\right)},\forall n=1,\ldots ,\bar{n}, \end{array}$$

(9) $$\begin{array}{cc} y_{1,n}^{\left(2\right)} & ={h_{1,n}^{\left(2\right)}}^{H}v_{n}+\epsilon_{1,n}^{\left(2\right)},\forall n=1,\ldots ,\bar{n}. \end{array}$$

Each user achieves an interference-free stream in every channel use, yielding the maximum sum-DoF of $d_{1}+d_{2}=2$, corresponding to the vertex $\left(1,1\right)$.

### 3.2. Time Division Multiple Access

Without any CSIT, interference alignment is impossible. The only viable strategy is to use an orthogonal scheme, such as TDMA, which relies on orthogonal resource allocation in the time domain.

The scheme spans one time interval (T=1), which has $n_{1}=2\bar{n}$ channel uses. User 1 is allotted the first *n* channel uses (u is sent), and user 2 the remaining *n* uses (v is sent). The transmitted signal is $x_{1}={\left[u^{T},v^{T}\right]}^{T}\in C^{2M\bar{n}}$. Since there is no CSIT, no precoding is applied, i.e., no SIA. Since the total time duration is $2\bar{n}$ uses, and each user is allocated *n* interference-free uses. The DoF is $d_{1}=d_{2}=\frac{1}{2}$, yielding a sum-DoF of 1.

### 3.3. Maddah-Ali and Tse (MAT) Scheme

The MAT scheme [10] utilizes perfect delayed CSIT (with no current CSIT, α=0) to achieve TIA across T=2 time intervals.

The scheme uses $n_{1}=2\hat{n}$ channel uses in time interval t=1 (TDMA-like orthogonal transmission) and $n_{2}=\hat{n}$ channel uses in time interval t=2 for the retrospective IA signal, an interference-resolving signal designed to recover the interference-corrupted messages from the previous interval. In t=2, the BS sends $x_{2,n}={h_{1,n}^{\left(2\right)}}^{H}u_{n}+{h_{1,\hat{n}+n}^{\left(1\right)}}^{H}v_{n},$$\;\forall n=1,\ldots ,\hat{n}$, which is the sum of the previously overheard interferences using the known delayed channel state. For $\hat{n}=1$, the received signal at user 1 is as follows:

(10) $$\left[\begin{array}{c} y_{1,1}^{\left(1\right)}\\ y_{1,2}^{\left(1\right)}\\ y_{2,1}^{\left(1\right)} \end{array}\right]=\underset{\mathrm{rank}=2}{\underset{︸}{\left[\begin{array}{c} {h_{1,1}^{\left(1\right)}}^{H}\\ 0\\ {\left[h_{2,1}^{\left(1\right)}\right]}_{1}^{*}{h_{1,1}^{\left(2\right)}}^{H} \end{array}\right]}}u+\underset{\mathrm{rank}=1}{\underset{︸}{\left[\begin{array}{c} 0\\ {h_{1,2}^{\left(1\right)}}^{H}\\ {\left[h_{2,1}^{\left(1\right)}\right]}_{1}^{*}{h_{1,2}^{\left(1\right)}}^{H} \end{array}\right]}}v+\left[\begin{array}{c} \epsilon_{1,1}^{\left(1\right)}\\ \epsilon_{1,2}^{\left(1\right)}\\ \epsilon_{2,1}^{\left(1\right)} \end{array}\right]$$

The interference v is aligned qith a 1-D subspace, while the desired signal u spans a 2-D subspace. Since the total time is 3 channel uses, the DoF achieved by each user is $d_{1}=d_{2}=\frac{2}{3}$, with a sum-DoF of $\frac{4}{3}$.

The MAT variant [11] achieves the same DoF by sending $x_{1}=u+v$ (superposition in $n_{1}=\hat{n}$ channel uses) and sending the overheard interferences orthogonally in $n_{2}=2\hat{n}$ channel uses.

### 3.4. Rate-Splitting

The rate-splitting (RS) scheme [11] (Lemma 2) utilizes a combination of SIA and MIA to manage the imperfect current CSIT (0<α<1) over a single time interval (T=1).

The user messages are split into private and common parts, where the common parts are encoded into the same signal and must be decoded by all users. The transmitted signal $x_{1}=x_{c}+x_{p}^{\left(1\right)}+x_{p}^{\left(2\right)}$ consists of common ($x_{c}$) and private ({$x_{p}^{\left(k\right)}{\}}_{k=1}^{2}$) streams.

- SIA: The private streams are precoded with power $P_{p}$∼$P^{\alpha }$, using the imperfect current CSIT, such that unintended private interference is suppressed to the noise level.
- MIA: The common stream, with power $P_{c}$∼*P*, absorbs the residual interference components that cannot be accurately nulled due to CSIT uncertainty, thereby preventing the rate-saturation typical of conventional precoding in interference-limited regimes.

Decoding is based on SIC, where the common message is decoded first, and the unintended private message is treated as noise. The common message decoding achieves a DoF of $d_{c}=1-\alpha$. After cancellation, the private streams achieve DoF $d_{p,k}=\alpha$. This yields the total sum-DoF of $d_{1}+d_{2}=1+\alpha$, achieving the asymmetric DoF vertices $\left(1,\alpha \right)$ or $\left(\alpha ,1\right)$.

### 3.5. Time-Correlated Scheme

The time-correlated scheme (the term *time-correlated scheme* is adopted here to refer to the strategy proposed in [11] (Section IV-B), as it was not named in the original literature; this name reflects the exploitation of temporal channel correlation to enhance current CSIT via delayed feedback for multi-slot interference management) [11] (Section IV-B) is a space–time interference alignment (STIA) scheme designed for the scenario of perfect delayed CSIT and imperfect current CSIT (0<α<1). It serves as a combination of SIA and TIA, operating over two time intervals (T=2) to manage interference as follows:

- Time Interval t=1: User messages $w_{1}^{\left(1\right)}$ and $w_{1}^{\left(2\right)}$ are encoded and precoded into $x_{1}$ using the available imperfect current CSIT, similar to standard space-division multiple access (SDMA). While SIA precoding attempts to minimize interference, the CSIT uncertainty results in residual interference at each receiver, which is subsequently addressed via the retrospective alignment in the next interval.
- Time Interval t=2: The transmitter utilizes the delayed CSIT from t=1 to precisely calculate the interference that was overheard by the users. This interference is quantized and encoded into a common message $w_{c,2}$. This common message is then transmitted alongside new private user messages $w_{2}^{\left(1\right)}$ and $w_{2}^{\left(2\right)}$ using SIA precoding based on the current CSIT of the second interval.
- Decoding Process: The receivers employ a two-stage decoding strategy. First, they decode the common message $w_{c,2}$ and the private messages $w_{2}^{\left(1\right)},w_{2}^{\left(2\right)}$ from the signal received at t=2. Subsequently, the information from $w_{c,2}$ is used to reconstruct and cancel the residual interference present in the signal received during t=1. This reduces the interference from the first interval retroactively over the entire two-slot duration.

By effectively coupling the two time intervals, this integrated approach achieves a symmetric sum-DoF of $\frac{4+2\alpha }{3}$. This corresponds to the symmetric DoF point $\left(d_{1},d_{2}\right)=\left(\frac{2+\alpha }{3},\frac{2+\alpha }{3}\right)$. This point represents the information–theoretic optimal trade-off between the immediate gains from SIA and the retrospective gains from TIA under mixed CSIT conditions.

## 4. End-to-End Deep-Learning Using Interference Alignment

This section introduces the architecture of the proposed E2E DL-based model, which is a joint autoencoder designed to realize and integrate the three interference alignment (IA) techniques: space-domain, time-domain, and message-domain IA, under variety of CSIT conditions. The *deep space-time-message interference alignment (Deep-STMIA)* architecture consists of a neural network-based transmitter (encoder) and multiple parallel receiver (decoder) networks, which are optimized jointly using a custom loss function.

### 4.1. Transmitter Architecture

**Figure 3:**
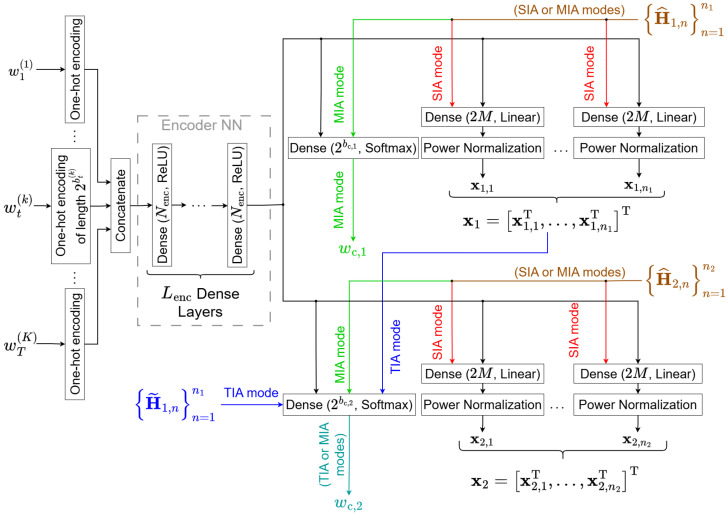
The Deep-STMIA transmitter block for T=2.

The transmitter architecture, illustrated in Figure 3, is designed to map the user messages and available CSIT into the spatio-temporal signal space. Specifically, it jointly generates the encoded signals and the common messages by processing the user messages alongside the current and delayed CSIT. This framework allows the model to E2E optimize the three IA techniques across all *T* time intervals.

The Deep-STMIA architecture performs joint processing across all time intervals. First, all user messages $w_{t}^{\left(k\right)},\forall k,\forall t$ are processed jointly by shared Encoder NN layers, which includes $L_{\mathrm{enc}}$ dense layers with $N_{\mathrm{enc}}$ neurons and ReLU activation shown in Figure 3. This joint approach offers two main benefits:

1. It ensures that the number of trainable parameters remains constant regardless of the number of time intervals *T*, enhancing scalability.
2. It allows for the design of transmit symbols, $x_{t}$, and common messages, $w_{c,t}$, to be jointly optimized over the entire sequence of *T* time intervals, facilitating the exploitation of inter-slot temporal dependencies. This is crucial for schemes using time-domain IA (TIA), where transmission in any given interval is coupled with the interference patterns of previous intervals.

The encoding process in Deep-STMIA is not restricted to standard constellation mapping. Instead, it utilizes a deep neural network to map messages into a high-dimensional feature space. This is analogous to the feature extraction process used in deep-learning-enabled semantic communication systems [20], enabling the transmitter to discover message representations that are inherently resilient to CSIT inaccuracies and facilitate more efficient interference alignment.

After initial message processing, the following final encoded signals and common messages are generated for the *T* time intervals via the following parallel layers:

- *Encoded Signal ($x_{t}$) Generation:* To ensure that the power constraint is satisfied on a per-symbol basis, the signal generated for each time interval *t* is achieved via a dedicated dense layer followed by a power normalization layer. Current CSIT (${\hat{H}}_{t,n},\forall n$), if available, is input to the corresponding dense layer processing encoded signal at time $t\in \left\{1,\ldots ,T\right\}$, allowing the network to learn a spatial precoding strategy for *space-domain interference alignment (SIA)*. This approach aligns with recent findings that deep learning-based beamformers can achieve superior robustness against CSI uncertainty compared to conventional iterative algorithms [22].
- *Common Message ($w_{c,t}$) Generation:* For the common message generation, rather than a discrete (binary bit) output, a probability vector of length $2^{b_{c,t}}$ is generated by a dense layer with a softmax activation function. This differentiable output facilitates the backpropagation process.
  **–**  MIA Implementation: The generation of $w_{c,t}$ is always based on the user messages. If only current CSIT is available, $w_{c,t}$ is conditioned on the user messages and ${\hat{H}}_{t,n},\forall n$.
  **–**  TIA Implementation: If delayed CSIT is accessible for t≥2, the common message generated at time $t\in \left\{2,\ldots ,T\right\}$ also uses the delayed CSIT (${\tilde{H}}_{t-1,n},\forall n$) and the encoded signal from the previous time interval ($x_{t-1}$) as inputs. This dependency allows the common message to contain the necessary retrospective alignment information for *time-domain interference alignment (TIA)*.

### 4.2. Receiver Architecture

**Figure 4:**
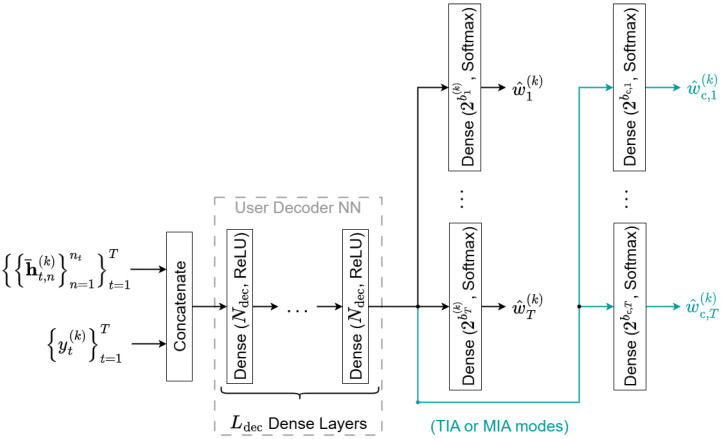
The Deep-STMIA decoder block at user $k\in \left\{1,\ldots ,K\right\}$ is represented.

The decoder block of the Deep-STMIA architecture shown in Figure 1 comprises *K* parallel user decoders. Each user *k* is responsible for decoding its private messages (${\hat{w}}_{t}^{\left(k\right)},\forall t$) and the common messages (${\hat{w}}_{c,t}^{\left(k\right)},\forall t$) for all time intervals, based on its received signals ($y_{t}^{\left(k\right)},\forall t$) and its local CSIR (${\bar{h}}_{t,n}^{\left(k\right)},\forall n,\forall t$), as illustrated in Figure 4.

Similarly to the transmitter, to maintain parameter efficiency and enable joint processing, received signals and local CSIR across all *T* time intervals are fed simultaneously into shared User Decoder NN layers, which include $L_{\mathrm{dec}}$ dense layers with $N_{\mathrm{dec}}$ neurons and ReLU activation, as shown in Figure 4. The decoder network learns the necessary interference cancellation and decoding logic over the entire time block.

Following the shared User Decoder NN, dedicated dense layers, each with softmax activation, are used to estimate the probability vector for each private message ${\hat{w}}_{t}^{\left(k\right)}$ and common message ${\hat{w}}_{c,t}^{\left(k\right)}$ for all t=1,…,T.

### 4.3. Implementation of Interference Alignment Techniques

The proposed Deep-STMIA architecture enables the use of space-domain and message-domain IA through specific signal and CSIT dependencies:

- Space-Domain IA (SIA) is implemented by feeding the current CSIT (${\hat{H}}_{t,n},\forall n$) as an input to the layers that determine the encoded signal $x_{t}$ in each time interval as shown in Figure 3. The network learns to compute the spatial precoding vectors based on instantaneous channel conditions.
- Message-Domain IA (MIA) is enabled by the common message $w_{c,t},\forall t$, which acts as a regularizer, determining the portion of information decoded by all users (rate-splitting concept [1]). The quality of current CSIT directly influences the common message generated, as less perfect CSIT necessitates a larger common stream for robustness. The common message must be generated at the transmitter and decoded at the receiver for MIA to be effective.
- Time-Domain IA (TIA) is realized when the common message generator at time t≥2 accepts the delayed CSIT and the previous time’s encoded signal ($x_{t-1}$) as input. This allows the common message to carry retrospective alignment information based on past channel conditions, incorporating the principles of the MAT scheme [10].

The synergy between these IA modes is visually represented in the architectural diagrams given by Figure 1, Figure 3 and Figure 4 as follows:

- SIA mode (red color).
- MIA mode (green color).
- TIA mode (blue color).
- The combination of SIA and/or MIA modes (brown color) shows current CSIT dependency.
- The combination of MIA and/or TIA modes (blue-green color) shows common message generation/decoding.

### 4.4. Training and Loss Function

The E2E system is trained by treating it as an autoencoder, where input messages are reconstructed at the output. The training process involves defining a custom loss function and specific hyperparameters, as summarized in Table 2. The goal of the training process is to minimize the total weighted loss function *L* (given by Equation (13) later on) with respect to the trainable parameters of the neural networks, θ.

Optimization is performed using the Adam optimizer [23], which adaptively adjusts the learning rate for each parameter, to achieve faster convergence. The learning rate in the first half of the epochs is 0.005 and then reduced to 0.001. Training is based on a data set of 100,000 input message combinations (samples). To handle this large dataset efficiently and enable stochastic gradient descent, the training is performed iteratively using a mini-batch size of 1024. The entire data set is passed through the network 30 times, which is the number of epochs. The system is trained at a high training SNR of 20 dB, which is increased to 30 dB in scenarios with intensified multi-user interference, such as those characterized by higher CSIT uncertainty. This strategy forces the network to learn efficient precoding and decoding strategies primarily limited by channel imperfections, i.e., errors in parameter estimation, quantization, and delay, rather than by noise, consistent with the objective of interference alignment.

The system includes auxiliary messages ($w_{c,t},\forall t$) that enhance robustness against imperfect CSIT by serving as a regularization mechanism in the deep learning model [24]. This regularization forces the network to learn features that are invariant to small perturbations in the channel state, thereby enhancing the model’s generalization to channel realizations not seen in the training set. By incorporating the common message as an auxiliary task, the regularization guides the optimizer toward more stable local minima, preventing the transmitter from over-optimizing beamforming vectors for noisy channel estimates. The above mechanism enhances reliability under imperfect CSIT. The loss function comprises the following:

- Private Message Loss ($L_{t}^{\left(k\right)},\forall k,\forall t$): Since user messages are represented by one-hot encoding, the loss for each message is calculated using the categorical cross-entropy function. Let $p_{t}^{\left(k\right)}$ denote the one-hot encoded ground truth vector of length $2^{b_{t}^{\left(k\right)}}$ and ${\hat{p}}_{t}^{\left(k\right)}$ denote the predicted probability vector for user *k* at time *t*. The loss is defined as follows:
  (11) $$L_{t}^{\left(k\right)}\left(p_{t}^{\left(k\right)},{\hat{p}}_{t}^{\left(k\right)}\right)=-\sum_{j=1}^{2^{b_{t}^{\left(k\right)}}}{\left[p_{t}^{\left(k\right)}\right]}_{j}\log\left({\left[{\hat{p}}_{t}^{\left(k\right)}\right]}_{j}\right).$$
- Common Message Loss ($L_{c,t}^{\left(k\right)},\forall k,\forall t$): As the common message generator outputs a probability vector and the decoder reconstructs a probability vector, the loss is computed using the Kullback–Leibler (KL) Divergence. For the common message distribution $q_{t}$ of length $2^{b_{c,t}}$ and its reconstruction ${\hat{q}}_{t}^{\left(k\right)}$ at receiver *k*, the loss is
  (12) $$L_{c,t}^{\left(k\right)}(q_{t}\left|\right|{\hat{q}}_{t}^{\left(k\right)})=\sum_{j=1}^{2^{b_{c,t}}}{\left[q_{t}\right]}_{j}\log\left(\frac{{\left[q_{t}\right]}_{j}}{{\left[{\hat{q}}_{t}^{\left(k\right)}\right]}_{j}}\right).$$
- Total Common Message Loss: Each common message must be decoded by all users. Therefore, the loss for a given common message $w_{c,t}$ is defined as the worst-case loss among all users: $L_{c,t}=\max_{k=1,\ldots ,K}L_{c,t}^{\left(k\right)},\forall t$.

The total loss function is a weighted sum of the user message losses and the common message losses:

(13) $$\begin{array}{cc} L & =\sum_{k=1}^{K}\mu^{\left(k\right)}\sum_{t=1}^{T}L_{t}^{\left(k\right)}+\mu_{c}\sum_{t=1}^{T}\max_{k=1,\ldots ,K}L_{c,t}^{\left(k\right)}, \end{array}$$

where $\mu^{\left(k\right)},\forall k=1,\ldots ,K,$ are the weights for each user’s loss to account for the multi-objective nature of the problem, and $\mu_{c}$ is the weight for the common message loss. A higher value of $\mu_{c}$ increases the emphasis on the auxiliary common message, thus strengthening the regularization effect. The optimal set of weights is typically determined via hyperparameter optimization.

To maintain fairness among users and prevent the system from solely minimizing the loss of a subset of users, *dynamic weighting* is applied to update the weights after each mini-batch. The dynamically weighted loss function in the *i*-th mini-batch is given by

(14) $$\begin{array}{cc} \tilde{L}\left(\theta_{i}\right) & =\sum_{k=1}^{K}\mu^{\left(k\right)}\sum_{t=1}^{T}\omega_{t}^{\left(k\right)}\left(\theta_{i-1}\right){\tilde{L}}_{t}^{\left(k\right)}\left(\theta_{i}\right)+\mu_{c}\sum_{t=1}^{T}\omega_{c,t}\left(\theta_{i-1}\right)\max_{k=1,\ldots ,K}{\tilde{L}}_{c,t}^{\left(k\right)}\left(\theta_{i}\right), \end{array}$$

where $\theta_{i}$ is the set of trainable parameters in the *i*-th mini-batch. The normalized dynamic weights are calculated as follows:

(15) $$\begin{array}{cc} \; & \omega_{t}^{\left(k\right)}\left(\theta_{i-1}\right)=\frac{\mu^{\left(k\right)}{\tilde{L}}_{t}^{\left(k\right)}\left(\theta_{i-1}\right)}{\Omega_{i-1}}, \end{array}$$

(16) $$\begin{array}{cc} \; & \omega_{c,t}\left(\theta_{i-1}\right)=\frac{\mu_{c}{\tilde{L}}_{c,t}\left(\theta_{i-1}\right)}{\Omega_{i-1}}, \end{array}$$

(17) $$\begin{array}{cc} \; & \Omega_{i}=\sum_{t=1}^{T}\sum_{k=1}^{K}\mu^{\left(k\right)}{\tilde{L}}_{t}^{\left(k\right)}\left(\theta_{i}\right)+\sum_{t=1}^{T}\mu_{c}\max_{k=1,\ldots ,K}{\tilde{L}}_{c,t}^{\left(k\right)}\left(\theta_{i}\right). \end{array}$$

Note that $\tilde{L}\left(\theta_{i}\right)$ represents the loss evaluated on a specific mini-batch $\theta_{i}$, whereas *L* represents the expected loss. The above mechanism ensures that training attention is adaptively allocated toward the messages or users currently exhibiting the highest loss, promoting more balanced training and improved overall fairness.

### 4.5. Complexity

The total inference complexity of Deep-STMIA is the sum of the operations across its functional blocks. For a fully connected (Dense) layer with *I* inputs and *O* outputs, the number of parameters, weights and biases, is (I×O)+O, and the number of floating-point operations (FLOPs) is 2×I×O, where each multiply-accumulate counts as 2 FLOPs. Below, the FLOP counts required for each component are detailed:

1. Transmitter Complexity ($C_{\mathrm{Tx}}=C_{\mathrm{Tx},1}+C_{\mathrm{Tx},2}+C_{\mathrm{Tx},3}$): The transmitter complexity is the summation of the flops required for (i) processing raw one-hot messages by Encoder NN ($C_{\mathrm{Tx},1}$), (ii) generating common messages ($C_{\mathrm{Tx},2}$), and (iii) generating the encoded signals ($C_{\mathrm{Tx},3}$), which are calculated as follows:
  (18) $$\begin{array}{cc} C_{\mathrm{Tx},1} & =2\sum_{t=1}^{T}\sum_{k=1}^{T}2^{b_{t}^{\left(k\right)}}N_{\mathrm{enc}}+2\left(L_{\mathrm{enc}}-1\right)N_{\mathrm{enc}}^{2}, \end{array}$$
  (19) $$\begin{array}{cc} C_{\mathrm{Tx},2} & =2^{b_{c,1}+1}\left(N_{\mathrm{enc}}+2MKn_{1}\right)+\sum_{t=2}^{T}2^{b_{c,t}+1}\left(N_{\mathrm{enc}}+2MKn_{t}+2Mn_{t-1}\right), \end{array}$$
  (20) $$\begin{array}{cc} C_{\mathrm{Tx},3} & =\sum_{t=1}^{T}4Mn_{t}\left(N_{\mathrm{enc}}+2MKn_{t}\right). \end{array}$$
2. Receiver Complexity ($C_{\mathrm{Rx}}=C_{\mathrm{Rx},1}+C_{\mathrm{Rx},2}+C_{\mathrm{Rx},3}$): The decoder at each user is designed to be lightweight, involving only the received signal processing and bit estimation. The FLOPs required for the *K* receivers is the summation of the FLOPs required for (i) processing CSIR and received signals by User Decoder NN ($C_{\mathrm{Rx},1}$), (ii) estimating common messages ($C_{\mathrm{Rx},2}$), and (iii) estimating user messages ($C_{\mathrm{Rx},3}$), which are as follows:
  (21) $$\begin{array}{cc} C_{\mathrm{Rx},1} & =2K\left(\sum_{t=1}^{T}2Mn_{t}+\sum_{t=1}^{T}2n_{t}\right)N_{\mathrm{dec}}+2K\left(L_{\mathrm{dec}}-1\right)N_{\mathrm{dec}}^{2}, \end{array}$$
  (22) $$\begin{array}{cc} C_{\mathrm{Rx},2} & =\sum_{k=1}^{K}\sum_{t=1}^{T}N_{\mathrm{dec}}2^{b_{c,t}+1}, \end{array}$$
  (23) $$\begin{array}{cc} C_{\mathrm{Rx},3} & =\sum_{k=1}^{K}\sum_{t=1}^{T}N_{\mathrm{dec}}2^{b_{t}^{\left(k\right)}+1}. \end{array}$$

The baseline schemes utilizing linear precoding (e.g., ZF-based RSMA) require the computation of precoding matrices based on the current CSIT. This typically involves a matrix inversion or a pseudo-inverse, with a complexity of approximately $O(M^{3})$. In contrast, the Deep-STMIA transmitter architecture consists of dense layers where the complexity scales as O(M) for each antenna element. This shift from cubic to linear scaling with respect to the number of antennas *M* suggests that Deep-STMIA is well-suited for future massive MISO systems.

As noted in (18)–(23), the current use of one-hot encoding leads to an exponential scaling with the number of users *K* (or information bits $b_{t}^{\left(k\right)}$). While this is manageable for the short-packet communications considered in this paper, this complexity can be further reduced to polynomial scaling by adopting bit-level message representations (e.g., binary cross-entropy loss on bit-strings).

## 5. Simulation Results

This section evaluates the block error rate (BLER) performance of the proposed Deep-STMIA framework. We consider a two-user MISO broadcast channel (M=K=2) and compare the proposed model with five baseline schemes that employ conventional interference alignment techniques, as detailed in Section 3. Each baseline represents an integrated E2E design comprising a specific combination of modulation, error-correction coding, precoding, interference alignment, and detection. To ensure a fair comparison, Deep-STMIA and the baselines are evaluated under identical user rates and CSIT conditions. If the number of time intervals, *T*, exceeds one, there is delayed CSIT. To allow for time-varying channels, delayed CSIT is limited to one previous time interval. Consequently, T=1 denotes the absence of delayed CSIT, whereas T=2 denotes that delayed CSIT is available from the previous time interval.

### 5.1. System Configuration

Without loss of generality, symmetric traffic is assumed, where each user receives the same number of information bits per time interval, i.e., $b_{t}^{\left(k\right)}=b_{t},\forall k,\forall t$. The system rate is characterized by the pair $\left(\bar{n},\bar{b}\right)$, representing the ratio of total channel uses to information bits per user over *T* intervals. Specifically, $\bar{n}=\sum_{t=1}^{T}n_{t}$ is the total channel uses, and $\bar{b}=\sum_{t=1}^{T}b_{t}$ is the total number of information bits per user. The effective transmission rate of a user is $R=\bar{b}/\bar{n}$ bits per channel use (bpcu).

Baselines utilize BPSK, 4-QAM, and 16-QAM. Error correction is implemented via a Hamming (7,4) code (also used in [17]), where each 7-bit codeword is zero-padded to 8 bits and mapped to two 16-QAM symbols. At the receiver, decoders employ soft-decision maximum likelihood (ML) detection:

(24) $$\begin{array}{c} \hat{c}=\arg\min_{c_{i}\in C}{∥r-c_{i}∥}^{2}, \end{array}$$

where C is the codebook and *r* is the processed signal. Hard-decision Hamming decoding is also included for comparison. The SNR per channel use is related to $E_{b}/N_{0}$ by $P_{t}=\frac{E_{b}}{N_{0}}\frac{b_{t}}{n_{t}}$, where $E_{b}$ is the energy per bit, and $N_{0}$ is the noise power spectral density ratio. It is important to note that Deep-STMIA requires only local CSIR at the users, in contrast to several of the baseline schemes that require global CSIR. Also, in the simulations, this local CSIR is assumed to be perfect (γ=1).

### 5.2. Hyperparameter Settings and Regularization

The performance of the Deep-STMIA network is governed by the hyperparameters $L_{\mathrm{enc}}$, $L_{\mathrm{dec}}$, and the common bits $b_{c,t}$. The NN layer counts $L_{\mathrm{enc}}$ and $L_{\mathrm{dec}}$ define the processing capacity of the transmitter and decoders, respectively. In the design, layer counts are balanced. Otherwise, an excessively powerful decoder may learn to resolve interference via channel statistics obtained from backpropagation rather than instantaneous CSIT, resulting in a *lazy* transmitter that fails to exploit precoding gains. To ensure generalizability, the E2E processing capacity is calibrated to prevent *undercomplete* or *overcomplete* representations [25]. In the simulations, each dense layer in the encoder and decoder consists of $N_{\mathrm{enc}}=64$ and $N_{\mathrm{dec}}=64$ neurons with ReLU activation.

Auxiliary common bits $b_{c,t}$ provide structural regularization. While unnecessary under high-quality CSIT, they become critical as CSIT degrades, forcing the network to learn features invariant to channel state perturbations.

### 5.3. Extreme CSIT Regimes

The five baseline groups are categorized by the CSIT conditions defined in Section 3. The first three groups, which do not include an imperfect CSIT condition, serve as validation benchmarks to establish the reliability of Deep-STMIA in extreme CSIT regimes.

**Figure 5:**
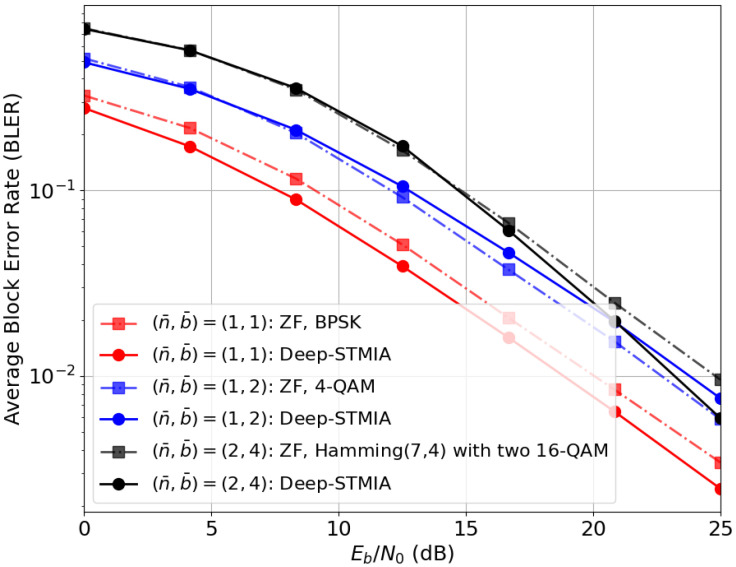
BLER performance comparison between the proposed Deep-STMIA ($L_{\mathrm{enc}}=3$, $L_{\mathrm{dec}}=1$, and $b_{c,1}=0$) and the ZF baseline under perfect current CSIT (α=1) for various $\left(\bar{n},\bar{b}\right)$ rate pairs.

(i) *Perfect Current CSIT:* In Figure 5, the BLER performance of Deep-STMIA is evaluated against $E_{b}/N_{0}$ under perfect current CSIT conditions (α=1) for three distinct rate pairs $\left(\bar{n},\bar{b}\right)$. For this scenario, the transmitter and user architectures are configured with $L_{\mathrm{enc}}=3$ and $L_{\mathrm{dec}}=1$ layers, respectively. Given the availability of perfect CSIT, message-domain regularization is unnecessary. Thus, $b_{c,1}$ is set to zero. Also, since time-domain interference management (TIA) is not required in the perfect CSIT regime, the number of time intervals is set to T=1.

As a benchmark, a conventional system employing ZF precoding is considered, which is known to achieve maximum DoF under perfect CSIT. The modulation and coding schemes for the baselines are selected to match the specific $\left(\bar{n},\bar{b}\right)$ pairs of the Deep-STMIA network. For instance, a message of $\bar{b}=4$ bits encoded via Hamming (7,4), zero-padded to 8 bits, and mapped to two 16-QAM symbols results in $\left(\bar{n},\bar{b}\right)=\left(2,4\right)$, yielding a rate of 2 bpcu per user. The results demonstrate that Deep-STMIA matches the performance of the ZF baseline and offers marginal gains for $\left(\bar{n},\bar{b}\right)=\left(1,1\right)$ and (1,2). This improvement is attributed to the framework’s ability to jointly optimize coding, modulation, and interference management in an E2E fashion.

**Figure 6:**
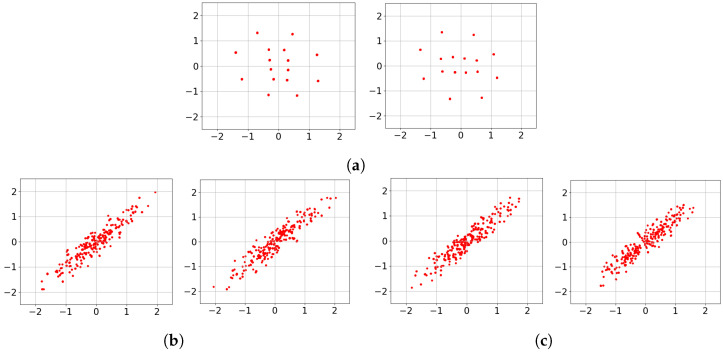
Learned transmit signaling of three values of $\left(\bar{n},\bar{b}\right)$ under perfect CSIT condition. (**a**) $\left(\bar{n},\bar{b}\right)=\left(1,2\right)$, Antenna 1 and 2. (**b**) $\left(\bar{n},\bar{b}\right)=\left(2,4\right)$, Antenna 1 and 2, n=1. (**c**) $\left(\bar{n},\bar{b}\right)=\left(2,4\right)$, Antenna 1 and 2, n=2.

To provide insight into the *black box* Deep-STMIA transmitter, the learned signaling under perfect CSIT is visualized in Figure 6. Each constellation is generated by an input of 500 samples to the trained transmitter, with training parameters matching the corresponding rates in Figure 5. For the case of $\left(\bar{n},\bar{b}\right)=\left(1,2\right)$ corresponding to Figure 6a, the learned constellations consist of well-separated $2^{K\bar{b}}=2^{4}$ regions. Notably, in Figure 6a, the second antenna constellation appears as a $90^{{}^{\circ}}$ rotation of the first, providing clear evidence of autonomously learned phase precoding across antenna elements.

**Figure 7:**
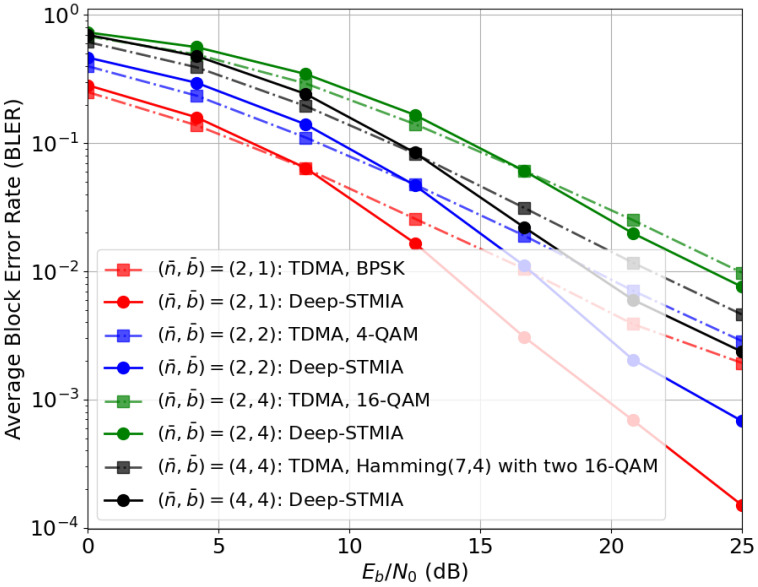
BLER performance comparison between Deep-STMIA ($L_{\mathrm{enc}}=3$, $L_{\mathrm{dec}}=1$, and $b_{c,1}=0$) and the TDMA baseline under the no-CSIT condition for various $\left(\bar{n},\bar{b}\right)$ pairs.

In contrast, for the higher rate of $\left(\bar{n},\bar{b}\right)=\left(2,4\right)$ shown in Figure 6b,c, the signaling no longer forms isolated regions and instead forms clouds along a stretched ellipse. This indicates that as transmission rate and spatio-temporal complexity increase, the Deep-STMIA framework shifts toward low-rank signaling in a specific spatial direction. This behavior suggests that under high interference, the model prioritizes interference alignment and spatial correlation over independent region separation. This shift in signaling strategy offers insight into why the BLER improvement over the Zero-Forcing baseline in Figure 5 is less pronounced for the (2,4) case compared to lower-rate configurations.

(ii) *No CSIT:* Figure 7 illustrates the BLER performance for the extreme case where the BS possesses no CSIT (neither current nor delayed). In this regime, a standard strategy for multi-user interference management is orthogonal transmission, such as TDMA, where each user is served once every *K* transmissions. For example, using 4-QAM modulation for $\bar{b}=2$ bits requires $\bar{n}=2$ channel uses per user (K=2), resulting in $\left(\bar{n},\bar{b}\right)=\left(2,2\right)$. To ensure a fair comparison, Deep-STMIA is evaluated using the same $\left(\bar{n},\bar{b}\right)$ values. Because the transmitter lacks CSIT, explicit SIA, MIA, and TIA strategies are not feasible. Hence, T=1 and $b_{c,1}=0$ are used.

The results in Figure 7 show that Deep-STMIA noticeably outperforms TDMA in the high-SNR regime, particularly for lower code rates. This suggests that the Deep-STMIA transmitter learns the underlying channel statistics through backpropagation during training, allowing it to move beyond the rigid orthogonality of TDMA and utilize the channel more effectively. Conversely, Deep-STMIA exhibits slightly lower performance than TDMA at low SNR. This can be attributed to the fact that Deep-STMIA utilizes all *n* channel uses for simultaneous transmission, whereas TDMA concentrates power into n/K uses. While TDMA benefits from higher symbol energy concentration, Deep-STMIA’s joint transmission is more susceptible to noise accumulation in power-limited regimes.

**Figure 8:**
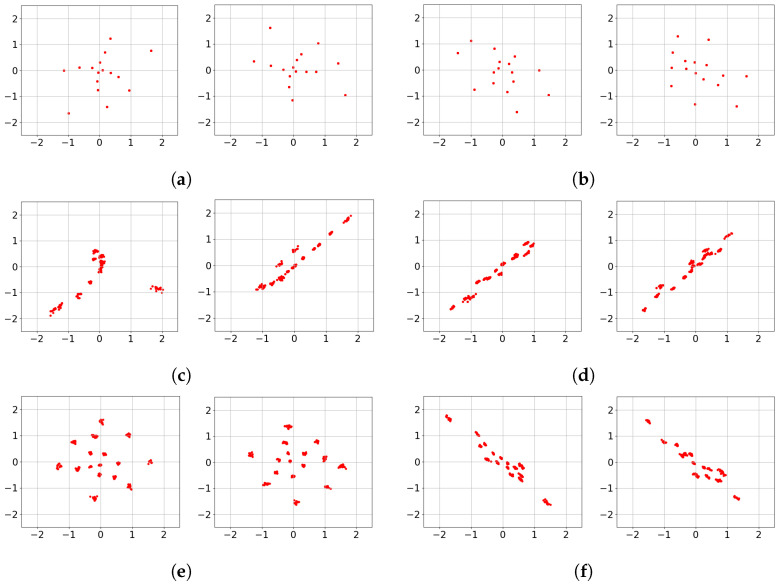
Learned transmit signaling of four values of $\left(\bar{n},\bar{b}\right)$ under no CSIT condition. (**a**) $\left(\bar{n},\bar{b}\right)=\left(2,2\right)$, Antenna 1 and 2, n=1. (**b**) $\left(\bar{n},\bar{b}\right)=\left(2,2\right)$, Antenna 1 and 2, n=2. (**c**) $\left(\bar{n},\bar{b}\right)=\left(4,4\right)$, Antenna 1 and 2, n=1. (**d**) $\left(\bar{n},\bar{b}\right)=\left(4,4\right)$, Antenna 1 and 2, n=2. (**e**) $\left(\bar{n},\bar{b}\right)=\left(4,4\right)$, Antenna 1 and 2, n=3. (**f**) $\left(\bar{n},\bar{b}\right)=\left(4,4\right)$, Antenna 1 and 2, n=4.

The learned transmit signaling under the no-CSIT condition is illustrated in Figure 8, corresponding to the rates evaluated in Figure 7. In Figure 8a,b, representing $\left(\bar{n},\bar{b}\right)=\left(2,2\right)$, the constellations across all channel uses consist of $2^{K\bar{b}}=2^{4}$ distinct point regions. This indicates that Deep-STMIA avoids a naïve TDMA approach; instead, it utilizes all available spatio-temporal resources for all user messages simultaneously, rather than employing orthogonal time division.

Consistent with the behavior observed in Figure 6, the constellations in Figure 8a,b exhibit clear phase precoding across antenna elements. Interestingly, these learned constellations appear to form halves of a symmetric geometric shape, suggesting the discovery of a sophisticated signaling partition in the complex plane rather than the time domain.

**Figure 9:**
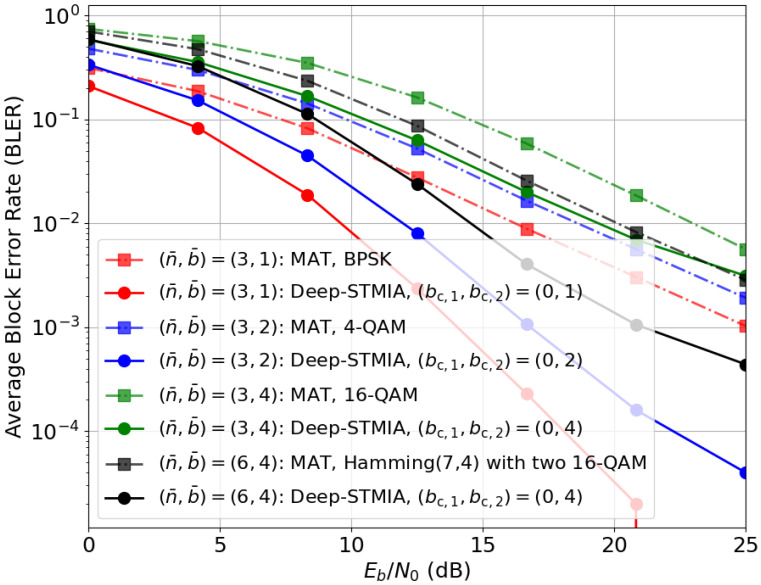
BLER performance of Deep-STMIA ($L_{\mathrm{enc}}=3$, $L_{\mathrm{dec}}=1$) compared to the MAT scheme [10] under perfect delayed CSIT ($\beta_{\mathrm{train}}=0.8$, β=1) and no current CSIT ($\alpha_{\mathrm{train}}=\alpha =0$) for T=2 time intervals.

For the higher complexity case of $\left(\bar{n},\bar{b}\right)=\left(4,4\right)$ shown in Figure 8c–f, the model shifts toward a time-division strategy. Each 2D constellation consists of only $2^{4}$ point regions, implying that half of the users’ bits are transmitted in the first two channel uses, while the remaining bits are sent in the subsequent two uses. This demonstrates that as spatio-temporal resources increase, Deep-STMIA tends toward orthogonal transmission rather than joint optimization across the entire resource block. This shift toward orthogonalization explains the less pronounced performance gains over the TDMA baseline observed for the higher rates in Figure 7.

(iii) *Perfect Delayed CSIT:* The third extreme scenario involves completely outdated CSIT, characterized by perfect delayed CSIT and a total absence of current CSIT, as evaluated in Figure 9. To facilitate interference management over time, the block length is set to T=2. The primary benchmark for this regime is the MAT scheme [10], which utilizes retrospective interference alignment. In the first time interval (t=1), MAT performs TDMA-style transmission. In the second interval (t=2), the transmitter uses delayed CSIT to reconstruct and transmit a superposition of the interference signals overheard by users, thus increasing the DoF relative to TDMA.

For a modulated signal of $\bar{b}$ bits requiring $\hat{n}$ channel uses, the MAT scheme requires $n_{1}=2\hat{n}$ channel uses in the first time interval and $n_{2}=\hat{n}$ uses in the second time interval, which is for transmission of the interference resolving signal, resulting in a total pair of $\left(\bar{n},\bar{b}\right)=\left(3\hat{n},\bar{b}\right)$. To ensure a fair comparison, the Deep-STMIA parameters for the two intervals are configured as $\left[\left(n_{1},b_{1}\right),\left(n_{2},b_{2}\right)\right]=\left[\left(2\hat{n},\bar{b}\right),\left(\hat{n},0\right)\right]$. Since current CSIT is not available at t=1, $b_{c,1}$ is set to zero. For t=2, the number of common bits is set to $b_{c,2}=\bar{b}$ to emulate the multicast nature of the second MAT phase. While the MAT scheme delivers these interference-resolving signals in an analog-domain superposition, Deep-STMIA treats them as a digital common message regularized to the user message size.

The results in Figure 9 demonstrate a significant performance improvement by Deep-STMIA compared to the conventional MAT scheme. Similarly to the no-CSIT case, this gain likely stems from the transmitter’s ability to learn and exploit channel statistics through the training process, allowing for more sophisticated joint processing across both time intervals. Overall, the competitive performance of Deep-STMIA under these extreme CSIT conditions confirms that the proposed system preserves the gains of established theoretical frameworks while validating its ability to autonomously learn effective communication strategies.

**Figure 10:**
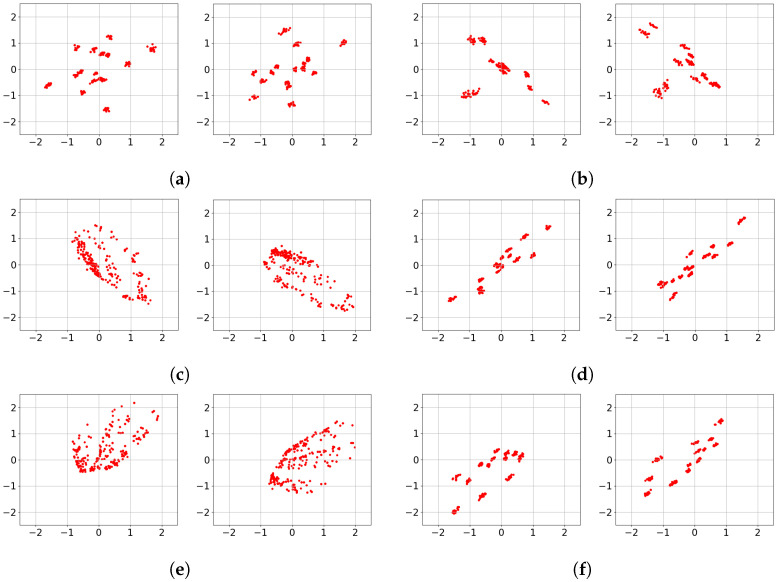
Learned transmit signaling of two values of $\left(\bar{n},\bar{b}\right)$ under perfect delayed CSIT and no current CSIT condition. (**a**) $\left(\bar{n},\bar{b}\right)=\left(6,4\right)$, Antenna 1 and 2, t=1,n=1. (**b**) $\left(\bar{n},\bar{b}\right)=\left(6,4\right)$, Antenna 1 and 2, t=1,n=2. (**c**) $\left(\bar{n},\bar{b}\right)=\left(6,4\right)$, Antenna 1 and 2, t=1,n=3. (**d**) $\left(\bar{n},\bar{b}\right)=\left(6,4\right)$, Antenna 1 and 2, t=1,n=4. (**e**) $\left(\bar{n},\bar{b}\right)=\left(6,4\right)$, Antenna 1 and 2, t=2,n=1. (**f**) $\left(\bar{n},\bar{b}\right)=\left(6,4\right)$, Antenna 1 and 2, t=2,n=2.

The learned signaling of Deep-STMIA under perfect delayed CSIT (with no current CSIT) is visualized in Figure 10 for the configuration $\left(\bar{n},\bar{b}\right)=\left(6,4\right)$. Under these conditions, the framework operates similarly to the no-CSIT case but leverages an additional time interval to transmit a common message that resolves interference generated during the first interval. Observations from Figure 10 reveal that the constellations in t=2 appear as phase-precoded versions of specific clusters from t=1. This suggests that the common message in the second time interval employs a retransmission strategy with optimized phase alignment, designed to coherently resolve multi-user interference based on the feedback of past channel states.

### 5.4. Practical CSIT Regime

The remaining two evaluation groups characterize the practical case of imperfect CSIT conditions.

**Figure 11:**
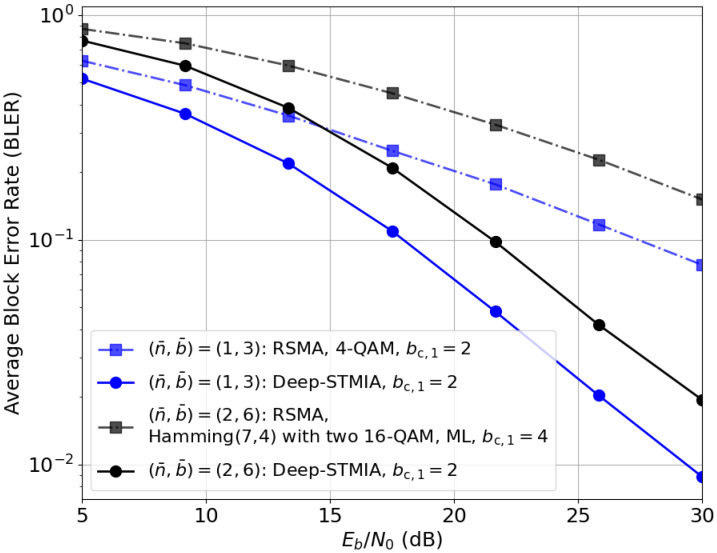
BLER performance for two values for $\left(\bar{n},\bar{b}\right)$ under imperfect current CSIT ($\alpha_{\mathrm{train}}=0.3$, α=0.5): Deep-STMIA ($L_{\mathrm{enc}}=3$ and $L_{\mathrm{dec}}=2$) vs. the RSMA baseline.

(i) *Imperfect Current CSIT:* Figure 11 illustrates the BLER performance of Deep-STMIA and its corresponding baseline, RSMA [1], for two rate pairs $\left(\bar{n},\bar{b}\right)$ under imperfect current CSIT (α=0.5) with no delayed CSIT. Since current CSIT is the only available information, the block length is set to T=1.

Rate-splitting is recognized as the DoF-optimal strategy for MISO BC under imperfect current CSIT [5]. In this scheme, each user message of $\bar{b}$ bits is split into $b_{p}$ private bits and $b_{c}/K$ common bits. The common bits from all users are aggregated into a single $b_{c}$-bit common message that must be decoded by all users. For instance, in a two-user system, a user message of $\bar{b}=3$ bits (comprising 2 private bits and 1 common bit) transmitted in $\bar{n}=1$ channel use results in a rate pair of (1,3). Here, $\bar{b}$ represents the total information bits per user, regardless of the private–common split.

While both schemes utilize common bits, the number of common bits for a given $\left(\bar{n},\bar{b}\right)$ may differ. For $\left(\bar{n},\bar{b}\right)=\left(2,6\right)$, the RSMA baseline utilizes four common bits, whereas Deep-STMIA achieves superior performance with only two common bits. This suggests that the E2E model uses the message-domain resources more efficiently. To address the increased complexity of multi-user interference management under imperfect CSIT, the decoder capacity is increased to $L_{\mathrm{dec}}=2$, empowering the users to better resolve the signals when the transmitter’s precoding gain is limited.

As shown in Figure 11, Deep-STMIA significantly outperforms RSMA for both values of $\left(\bar{n},\bar{b}\right)$. As highlighted in [26], SIC remains a core component and a limitation of RSMA. The observed performance gap suggests that while RSMA is theoretically robust, its practical link-level performance is hindered by the inherent error propagation in SIC decoding. In contrast, the neural network decoder can learn to map the composite received signal directly to message estimates, thereby mitigating the catastrophic effects of error propagation.

Table 3 presents the parameter counts and execution run time for Deep-STMIA and the RSMA baseline, corresponding to the scenarios evaluated in Figure 11. Both schemes were evaluated in a Google Colab environment utilizing an Intel Xeon CPU @ 2.20 GHz. While Deep-STMIA incurs a significant one-time offline training cost, the resulting model is highly efficient for online deployment. As shown in Table 3, the trained parameters (weights and biases) occupy approximately 20–30 KB of memory, a negligible hardware footprint. During inference, Deep-STMIA replaces computationally expensive analytical algorithms with simple feed-forward layers. These layers consist of multiply-accumulate operations that can be efficiently parallelized in hardware. In contrast, RSMA with linear precoding requires real-time matrix inversions with $O(M^{3})$ complexity for every channel update.

Deep-STMIA’s 10-fold speedup over RSMA is driven by its deterministic, feed-forward architecture, which is natively optimized for parallel processing (vectorization). Unlike traditional RSMA, which requires sequential and computationally expensive matrix inversions for every channel realization, Deep-STMIA processes large transmission blocks simultaneously. This architectural efficiency allows the proposed framework to achieve significantly higher computational throughput as measured on current hardware, making it a more feasible candidate for high-speed, low-latency 6G applications.

To evaluate the practical feasibility of Deep-STMIA, its run time must be considered in relation to the channel coherence time, $T_{c}$. In typical urban vehicular environments (e.g., v=50 km/h at 5.8 GHz), $T_{c}$ is approximately 5–10 ms. As shown in Table 3, Deep-STMIA achieves an average execution run time of 0.045 ms and 0.063 ms per sample for the considered scenarios. This provides significant computational headroom, ensuring that the inference is completed well within a coherence interval. In contrast, while the RSMA baseline also fits within these bounds for static or pedestrian speeds, its higher per-sample complexity (approx. 0.477 ms to 1.048 ms) leaves significantly less margin for other essential system tasks, such as channel estimation, particularly as carrier frequencies and user velocities increase in 6G standards.

**Figure 12:**
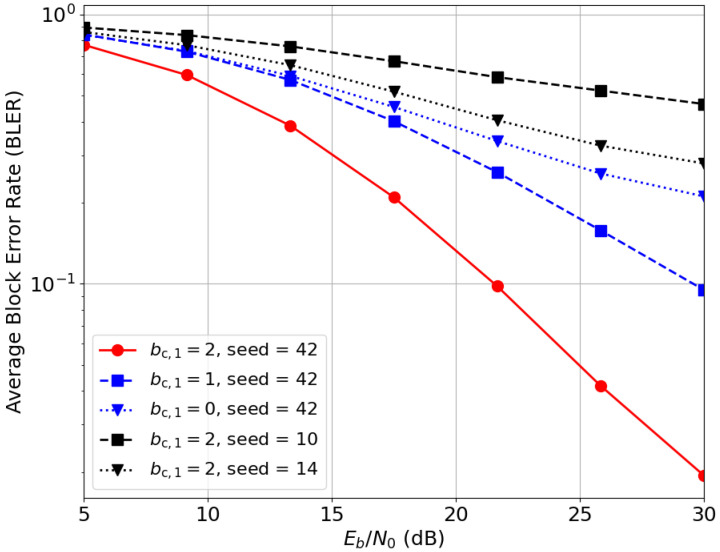
Deep-STMIA BLER performance for $\left(\bar{n},\bar{b}\right)=\left(2,6\right)$, $L_{\mathrm{enc}}=3$, and $L_{\mathrm{dec}}=2$ under imperfect current CSIT ($\alpha_{\mathrm{train}}=0.3$, α=0.5). The curves demonstrate the impact of different random seeds and common bit allocations.

While setting a fixed random seed ensures reproducibility, the non-convex nature of E2E optimization makes the deep learning model sensitive to initialization point when navigating a landscape with multiple local minima. To optimize performance, we evaluated several random seed values and present the results for the best-performing initialization (seed 42). Figure 12 illustrates this sensitivity, comparing the BLER performance for $\left(\bar{n},\bar{b}\right)=\left(2,6\right)$ under imperfect CSIT across three seeds (10, 14, and 42). Notably, seed 42 achieves a significantly lower BLER, indicating a more efficient signaling strategy. Figure 12 also demonstrates the impact of common message regularization: for $\left(\bar{n},\bar{b}\right)=\left(2,6\right)$, increasing the common bit allocation from $b_{c,1}=0$ to 2 results in a marked improvement in reliability. This suggests that for a fixed transmission rate, the allocation of common bits is crucial for mitigating channel uncertainty.

**Figure 13:**
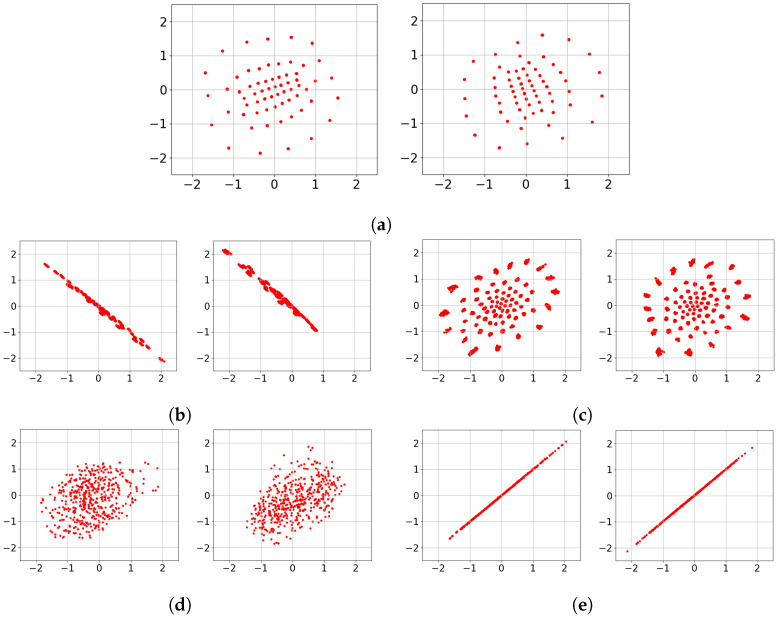
Learned transmit signaling of two values of $\left(\bar{n},\bar{b}\right)$ under imperfect current CSIT of quality α=0.5 and no delayed CSIT condition. The learnt signaling for $\left(\bar{n},\bar{b}\right)=\left(2,6\right)$ is represented for $b_{c,1}=2$ and 0. (**a**) $\left(\bar{n},\bar{b},b_{c,1}\right)=\left(1,3,2\right)$, Antenna 1 and 2. (**b**) $\left(\bar{n},\bar{b},b_{c,1}\right)=\left(2,6,2\right)$, Antenna 1 and 2, n=1. (**c**) $\left(\bar{n},\bar{b},b_{c,1}\right)=\left(2,6,2\right)$, Antenna 1 and 2, n=2. (**d**) $\left(\bar{n},\bar{b},b_{c,1}\right)=\left(2,6,0\right)$, Antenna 1 and 2, n=1. (**e**) $\left(\bar{n},\bar{b},b_{c,1}\right)=\left(2,6,0\right)$, Antenna 1 and 2, n=2.

The learned transmit signaling of Deep-STMIA under imperfect current CSIT (α=0.5) is illustrated in Figure 13 for rates $\left(\bar{n},\bar{b}\right)=\left(1,3\right)$ and (2,6). For the $\left(\bar{n},\bar{b}\right)=\left(1,3\right)$ case in Figure 13a, the framework discovers a well-separated 64-point constellation with evident phase precoding between the two antenna elements.

As the rate increases to (2,6), the signaling strategy becomes more sophisticated, as shown in Figure 13b–e. For both the $b_{c}=2$ (Figure 13b,c) and $b_{c}=0$ (Figure 13d,e) configurations, the model collapses the signaling in one channel use into a rank-one diagonal constellation, though the spatial directions differ. However, the signaling in the second channel use varies significantly based on the presence of common bits. While the $b_{c}=0$ case results in a cloud-like distribution of points, the $b_{c}=2$ case produces distinct, well-separated point regions (Figure 13c). Notably, the number of observed regions in Figure 13c is far fewer than $2^{12}$, confirming that the model prioritizes rank-one signaling in the first channel use. This indicates that Deep-STMIA autonomously ‘anchors’ the common bits to a robust rank-one structure to ensure successful decoding by both users under channel uncertainty. Conversely, the lack of well-separated regions in the $b_{c}=0$ case (Figure 13d,e) suggests that without common message regularization, the framework struggles to optimize the constellation geometry and directional alignment, thereby highlighting the critical role of common bits in stabilizing the learned signaling strategy.

**Figure 14:**
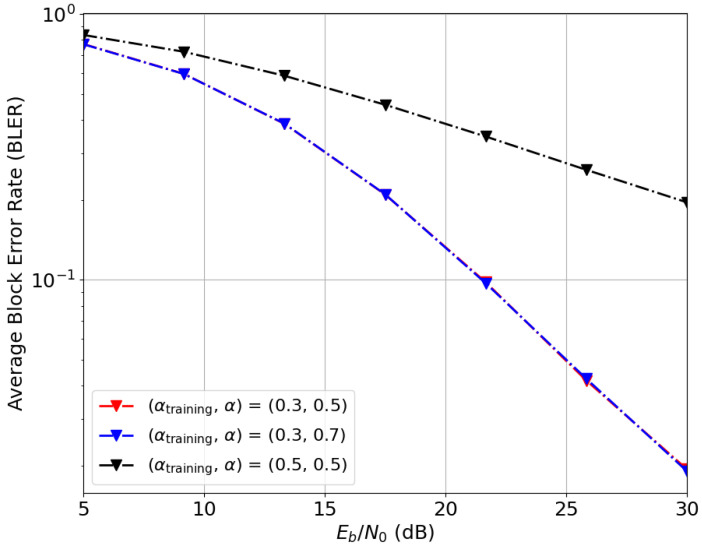
Deep-STMIA BLER performance for $\left(\bar{n},\bar{b}\right)=\left(2,6\right)$, $b_{c,1}=2$, $L_{\mathrm{enc}}=3$, and $L_{\mathrm{dec}}=2$ under imperfect current CSIT. Curves show the impact of current CSIT quality in training and evaluation phases. Note that the red and blue curves overlap.

**Figure 15:**
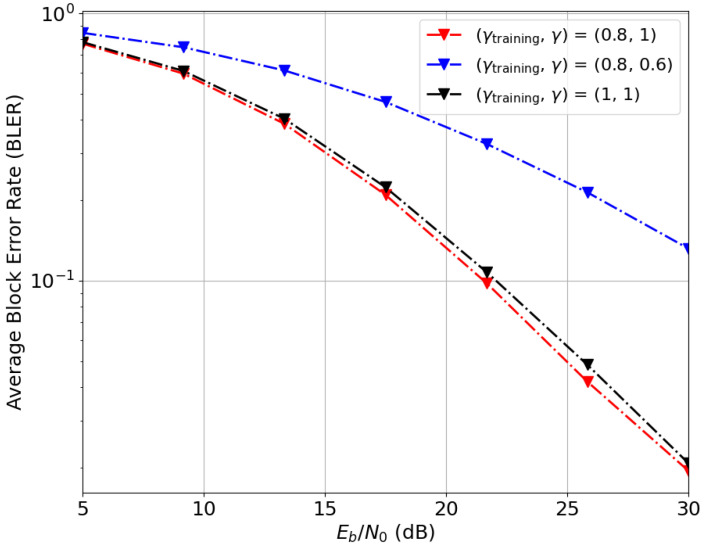
Deep-STMIA BLER performance for $\left(\bar{n},\bar{b}\right)=\left(2,6\right)$, $b_{c,1}=2$, $L_{\mathrm{enc}}=3$, and $L_{\mathrm{dec}}=2$ under imperfect current CSIT ($\alpha_{\mathrm{train}}=0.3,\alpha =0.5$). The curves demonstrate the impact of CSIR quality in training and evaluation phases.

Figure 14 and Figure 15 examine generalization to new samples and sensitivity to instantaneous CSI, the impact of current CSIT quality (α) and CSIR quality (γ) during both training and evaluation phases. The BLER performance for $\left(\bar{n},\bar{b}\right)=\left(2,6\right)$ is evaluated in Figure 14 for three $\left(\alpha_{\mathrm{train}},\alpha \right)$ pairs. Comparing the red and black curves, it is evident that BLER performance is heavily influenced by CSIT quality during the training phase, $\alpha_{\mathrm{train}}$. However, for a fixed $\alpha_{\mathrm{train}}$ (red and blue curves), CSIT quality during evaluation (α) has a negligible effect on performance. These results indicate that while CSIT quality significantly shapes the learned signaling strategy offline, the resulting precoder adopts a quasi-deterministic structure that does not rely on instantaneous CSIT feedback.

In Figure 15, the effect of CSIR quality is explored for three $\left(\gamma_{\mathrm{train}},\gamma \right)$ pairs. A comparison of the red and black curves shows that setting $\gamma_{\mathrm{train}}$ slightly lower than evaluation quality γ improves BLER performance and enhances generalization to new samples. In addition, comparing the red and blue curves that represent the same model evaluated under different CSIR qualities reveals that, unlike the trend observed for CSIT, the model is highly sensitive to instantaneous CSIR.

Consequently, system performance during inference is primarily governed by the receiver’s local channel knowledge. This suggests that the Deep-STMIA framework produces a robust blind precoding strategy, effectively decoupling the transmitter’s structural reliability from the volatility of instantaneous CSIT. In this configuration, the transmitter operates invariantly to CSIT after training, while the receiver uses local CSIR to unwarp the channel effects. This characteristic makes Deep-STMIA a highly suitable candidate for high-mobility or low-latency scenarios, where maintaining a stable link without constant, perfect feedback is critical.

Deep-learning-based E2E communication designs inherently encounter the curse of dimensionality [17]. In a *K*-user MISO BC system with a transmission rate of $\left(\bar{n},\bar{b}\right)$ per user, the number of information bits per interval of $\bar{n}$ channel uses is $K\bar{b}$, resulting in a codebook size of $2^{K\bar{b}}$ that grows exponentially with the number of users. However, as demonstrated in Figure 13b–e, common-message regularization assists Deep-STMIA in generating well-separated constellations with significantly fewer regions than the full codebook size by leveraging rank-one signaling structures. Thus, while common-message decoding may not entirely eliminate the dimensionality constraint, it effectively alleviates its severity.

**Figure 16:**
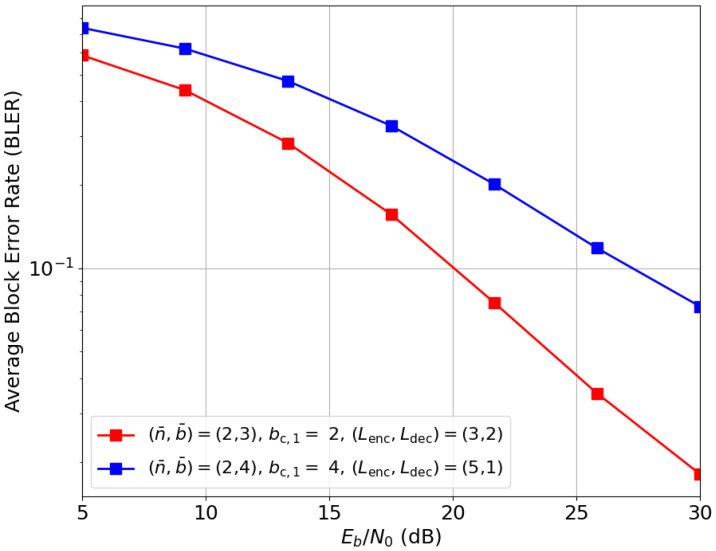
Deep-STMIA BLER performance for (M,K)=(4,4) under imperfect current CSIT ($\alpha_{\mathrm{train}}=0.3$, α=0.5).

To demonstrate the scalability of Deep-STMIA for higher values of *M* and *K*, Figure 16 presents the BLER performance for an (M,K)=(4,4) system under imperfect current CSIT ($\alpha_{\mathrm{train}}=0.3,\alpha =0.5$). Two rates are evaluated: $\left(\bar{n},\bar{b}\right)=\left(2,3\right)$ and (2,4), involving codebook sizes of $2^{12}$ and $2^{16}$, respectively. For the higher rate, the neural network (NN) depth is adjusted to $\left(L_{\mathrm{enc}},L_{\mathrm{dec}}\right)=\left(5,1\right)$, resulting in a total of $L_{\mathrm{dec}}K=4$ layers across all user decoders. These layer counts are selected to provide the transmitter with greater processing capacity than the receiver. This architectural imbalance is necessary to ensure the transmitter successfully learns to manage interference rather than becoming overly reliant on receiver-side processing.

**Figure 17:**
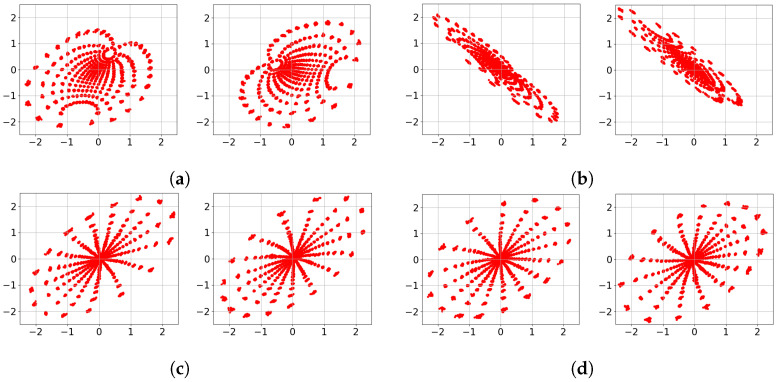
Learned transmit signaling constellations of two values of $\left(\bar{n},\bar{b}\right)$ for the system (M,K)=(4,4) under imperfect current CSIT of quality α=0.5 and no delayed CSIT. (**a**) $\left(\bar{n},\bar{b},b_{c,1}\right)=\left(2,4,4\right)$, Antenna 1 and 2, n=1. (**b**) $\left(\bar{n},\bar{b},b_{c,1}\right)=\left(2,4,4\right)$, Antenna 3 and 4, n=1. (**c**) $\left(\bar{n},\bar{b},b_{c,1}\right)=\left(2,4,4\right)$, Antenna 1 and 2, n=2. (**d**) $\left(\bar{n},\bar{b},b_{c,1}\right)=\left(2,4,4\right)$, Antenna 3 and 4, n=2.

**Figure 18:**
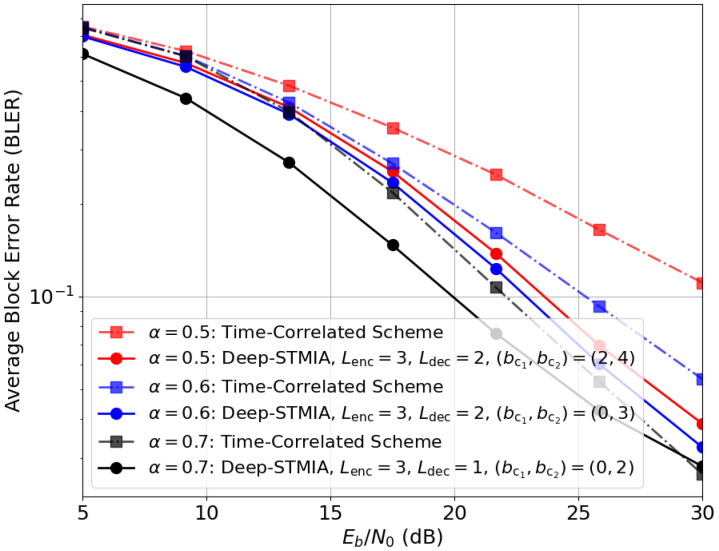
BLER performance under mixed CSIT (perfect delayed + imperfect current): Deep-STMIA vs. the time-correlated baseline for $\left(\bar{n},\bar{b}\right)=\left(3,8\right)$ and varying α.

The learned signaling for the rate $\left(\bar{n},\bar{b}\right)=\left(2,4\right)$ evaluated in Figure 16 is visualized in Figure 17, revealing well-separated constellation points despite the massive codebook sizes. Furthermore, Figure 17a,b demonstrate that the framework evolves beyond simple phase precoding; it utilizes joint phase and amplitude precoding across the four antenna elements. This highlights the capacity of Deep-STMIA to perform sophisticated interference alignment through multi-antenna coordination.

(ii) *Mixed CSIT (Imperfect Current + Perfect Delayed):* Lastly, Figure 18 evaluates the Deep-STMIA framework under the mixed CSIT regime (perfect delayed and imperfect current CSIT). To exploit the temporal degrees of freedom, the block length is set to T=2. In these simulations, the BLER is calculated based on blocks of 4 information bits. The rate pair is defined as $\left(\bar{n},\bar{b}\right)=\left(3,8\right)$, with the transmission is partitioned such that $\left(n_{1},b_{1}\right)=\left(1,4\right)$ and $\left(n_{2},b_{2}\right)=\left(2,4\right)$. The larger allocation of channel uses to the second interval ($n_{2}>n_{1}$) accommodates the retrospective interference-resolving signals. The system achieves a transmission rate of R=8/3≈2.67 bpcu per user.

As illustrated in Figure 18, Deep-STMIA consistently outperforms the time-correlated baseline [11]. In particular, as the current CSIT quality α improves, the model autonomously optimizes its internal representation, requiring fewer common bits $b_{c,t}$ for regularization. This suggests that the network effectively transitions its strategy from a MAT-like retrospective alignment to a more SIA-focused approach as the current CSIT becomes more reliable.

## 6. Conclusions

Deep-STMIA is introduced as an end-to-end deep-learning-based framework designed to manage multi-user interference in MU-MISO broadcast channels with imperfect current and delayed CSIT. By integrating space-, time-, and message-domain interference alignment into a unified neural network architecture, the limitations of conventional model-based schemes that rely on rigid decoding structures such as SIC can be improved upon. The findings indicate that the proposed framework not only matches the performance of classic theoretical benchmarks such as ZF and MAT in extreme CSIT scenarios but also provides substantial reliability gains in more practical imperfect current CSIT regimes.

A key contribution of Deep-STMIA is its ability to autonomously optimize message-domain regularization through auxiliary common bits, effectively generalizing the principles of RSMA while avoiding the pitfalls of error propagation. The simulation results underscore the robustness of the data-driven approach, showing that joint encoder–decoder design can effectively compensate for transmitter beamforming inaccuracies caused by CSI quantization and delay. Overall, Deep-STMIA provides a highly flexible and robust solution for interference management across a wide spectrum of CSIT conditions.

## Figures and Tables

**Table 1:** Comparison of sum-DoF for various schemes in a system with M=K=2.

Scheme	CSIT Condition	Sum-DoF
ZF	Perfect Current CSIT	2
TDMA	No CSIT	1
MAT	Perfect Delayed CSIT and No Current CSIT	$\frac{4}{3}$
Rate-Splitting (RS)	Imperfect α-CSIT (α∈[0,1])	1+α
Time-correlated scheme	Imperfect α-CSIT and Perfect Delayed CSIT	$\frac{4+2\alpha }{3}$

**Table 2:** Training parameters.

Parameter	Value
Optimizer	Adam
Learning Rate 1	0.005
Learning Rate 2	0.001
Number of samples	100,000
Mini-batch size	1024
Number of epochs	30
Training SNR	20 dB or 30 dB
User *k* Loss Weight	$\mu^{\left(k\right)}=1,\forall k$
Common Message Loss Weight	$\mu_{c}=0.5$
Random seed	42 (unless mentioned otherwise)
Training CSIR quality $\gamma_{\mathrm{train}}$	0.8 (unless mentioned otherwise)
Training delayed CSIT quality $\beta_{\mathrm{train}}$	0.8 (if delayed CSIT available)
Training current CSIT quality $\alpha_{\mathrm{train}}$	(Mentioned in the text)

**Table 3:** Computational complexity and run-time comparison.

Scenario, Scheme	Trainable Parameters	Execution Run Time Measured for 1000 Samples
(1,3,2), RSMA	—	0.477 s
(1,3,2), Deep-STMIA	20,768	0.045 s
(2,6,4), RSMA	—	1.048 s
(2,6,4), Deep-STMIA	36,372	0.063 s

## Data Availability

The simulation code for the Deep-STMIA framework is currently being organized for public release and will be hosted at https://github.com/ElahehSadeghabadi/Deep-STMIA (accessed on 30 December 2025). The complete repository will be populated with the finalized scripts upon publication.

## Abbreviations

The following abbreviations are used in this manuscript:

AWGN	Additive White Gaussian Noise
BS	Base Station
bpcu	bits per channel use
BLER	Block Error Rate
BC	Broadcast Channel
CSI	Channel State Information
CSIT	Channel State Information at the Transmitter
CSIR	Channel State Information at the Receiver
DL	Deep Learning
Deep-STMIA	Deep Space–Time-Message Interference Alignment
DoF	Degrees of Freedom
DPC	Dirty Paper Coding
E2E	End-to-End
FDD	Frequency-Division Duplexing
FLOP	FLoating-Point OPeration
i.i.d.	independent and identical distribution
IA	Interference Alignment
KL	Kullback–Leibler
MAT	Maddah-Ali and Tse
ML	Maximum Likelihood
MIA	Message-Domain Interference Alignment
MU-MISO	Multi-User Multiple-Input Single-Output
NR	New 5G Radio
RS	Rate-Splitting
RSMA	Rate-Splitting Multiple Access
SNR	Signal-to-Noise Ratio
SDMA	Space-Division Multiple Access
SIA	Space-Domain Interference Alignment
STIA	Space-Time Interference Alignment
SIC	Successive Interference Cancellation
TIA	Time-Domain Interference Alignment
TDMA	Time-Division Multiple Access
ZF	Zero Forcing
